# Prognostic value of serum nicotinamide phosphoribosyltransferase in patients with bladder cancer

**DOI:** 10.3325/cmj.2014.55.507

**Published:** 2014-10

**Authors:** Kui Zhang, Bin Zhou, Peng Zhang, Zhu Zhang, Peng Chen, Yan Pu, Yaping Song, Lin Zhang

**Affiliations:** 1 Department of Forensic Pathology, West China School of Preclinical and Forensic Medicine, Sichuan University, Chengdu, People’s Republic of China; 2 Laboratory of Molecular Translational Medicine, West China Institute of Women and Children's Health, West China Second University Hospital, Sichuan University, Chengdu, People’s Republic of China; 3 Department of Forensic Biology, West China School of Preclinical and Forensic Medicine, Sichuan University, Chengdu, People’s Republic of China; 4 Department of Urology, West China Hospital, Sichuan University, Chengdu, People’s Republic of China; 5 Department of Obstetrics and Gynecology, West China Second Hospital, Sichuan University, Chengdu, People’s Republic of China; The first two authors contributed equally to this work.

## Abstract

**Aim:**

To analyze the serum nicotinamide phosphoribosyltransferase (Nampt) level and its prognostic value in bladder cancer (BC).

**Methods:**

The study included 131 patients with transitional cell BC and 109 healthy controls from the West China Hospital of Sichuan University in the period between 2007 and 2013. Nampt concentration in serum was measured by commercial ELISA kits for human Nampt.

**Results:**

The serum Nampt protein level in patients with BC (mean ± standard deviation, 16.02 ± 7.95 ng/mL) was significantly higher than in the control group (6.46 ± 2.08 ng/mL) (*P* < 0.001). Serum Nampt level was an independent prognostic marker of non-muscle-invasive BC, with a higher serum Nampt level (>14.74 ng/mL) indicating shorter recurrence-free survival rate (hazard ratio = 2.85, 95% confidence interval, 1.01-8.06; *P* = 0.048).

**Conclusion:**

Our results suggest that serum Nampt level may serve as a biomarker of BC and an independent prognostic marker of non-muscle-invasive BC.

Bladder cancer (BC) is the ninth most common cancer diagnosis worldwide (1) and the most expensive cancer to treat (2). Among men it is the fourth most common cancer, with incidence four times higher than in women (3). In China, BC caused 17 365 deaths in 2005, with a steady increase in mortality between 1991 and 2005 (4). Of newly diagnosed BC cases, 70%-80% will present with non-muscle-invasive disease, 50%-70% will recur despite endoscopic and intravesical treatments, and 10%-30% will progress to muscle-invasive disease (5,6). Most recurrences occur within 5 years (7). Therefore, to develop improved, more effective prevention and treatments there is a need to find new biomarkers of tumorigenesis and prognosis of BC.

Nicotinamide phosphoribosyltransferase (Nampt) is a rate-limiting enzyme in the mammalian NAD^+^ biosynthesis of a salvage pathway (8). Previous studies have shown that it is significantly increased in primary colorectal cancer (9-11), lung cancer (12), breast cancer (13), prostate cancer (14) and gastric cancer (15). Thus, Nampt may be a good biomarker of malignant potential and stage progression (12,16). Our previous study revealed that genetic variants in *NAMPT* may predict BC risk and prognosis (17). In the present study, we analyzed the serum Nampt level and its prognostic value in BC.

## Materials and methods

### Study subjects

The study enrolled 131 patients with transitional cell BC (mean ± standard deviation [SD], 63.49 ± 12.96 years) and 109 healthy controls (63.12 ± 10.28 years) (17). All participants were recruited between 2007 and 2013 from the West China Hospital of Sichuan University. The diagnosis of BC was confirmed by histological tissue examination from biopsy or resected specimens. Seventy-two patients had muscle-invasive and 59 had non-muscle-invasive BC (Table 1). The control group consisted of 109 healthy individuals who underwent a routine health survey in the same hospital. We excluded those patients who had previous cancer, received radiotherapy or chemotherapy, had metastasized cancer from other or unknown origin, or had any other serious disease. Control subjects were genetically unrelated to the patients and to each other. All participants were Han Chinese living in Sichuan province of the southwest China. The study was approved by the hospital ethics committee and all participants gave a written informed consent.

**Table 1:** Clinicopathologic characteristics of 131 bladder cancer patients

Characteristics	Number of patients (%)
**Sex**	
Male	107 (81.7)
Female	24 (18.3)
**Age**	
≤64	57 (43.5)
>64	74 (56.5)
**Smoking**	
Smoking index >400	41 (31.3)
Smoking index ≤400	36 (27.5)
No	54 (41.2)
**Grade**	
Low	60 (45.8)
High	71 (54.2)
**Invasiveness**	
Muscle-invasive	72 (55.0)
Non-muscle-invasive	59 (45.0)
**Relapse**	
Yes	90 (68.7)
No	41 (31.3)
**Metastasis**	
Yes	20 (15.3)
No	111 (84.7)

### Investigation of plasma protein expression levels of Nampt

Serum was obtained after centrifuging blood samples at 1600 rpm for 10 min at 4°Cand stored at -80°C until analysis. Nampt concentration in serum was measured by commercial ELISA kits for human Nampt (USCNK, Wuhan, China). The antibodies used in ELISA are specific for human Nampt. Analytical limit of detection of Nampt was 6.3 pg/mL. The results are expressed in ng/mL.

### rs61330082 genotyping

*rs61330082,* a single-nucleotide polymorphism (SNP) in the promoter region of NAMPT gene, was genotyped by polymerase chain reaction (PCR)-restriction fragment length polymorphism (RFLP) method (17).

### Statistical analysis

Data were analyzed using SPSS for Windows software package, version 16.0 (SPSS Inc., Chicago, IL, USA). The difference in Nampt expression levels between BC patients and control group was tested by *t* test. The differences between serum Nampt level and genotypes of *rs61330082* were tested by one-way ANOVA analysis. Odds ratio (OR) and 95% confidence intervals (CI) were used to assess the effects of serum Nampt protein level on the clinicopathological factors.

Recurrence-free survival (RFS) and overall survival (OS) were estimated using the Kaplan-Meier method, and the association of Nampt expression with RFS and OS was examined by a log-rank test. OS was the time from the day of diagnosis to the day of death or the last follow-up. RFS was the time from the day of diagnosis to the day of recurrence or death or the last follow-up. Prognostic factors were determined using Cox regression analysis. *P* value ≤0.05 was considered statistically significant.

## Results

### Nampt expression level

**Figure 1:**
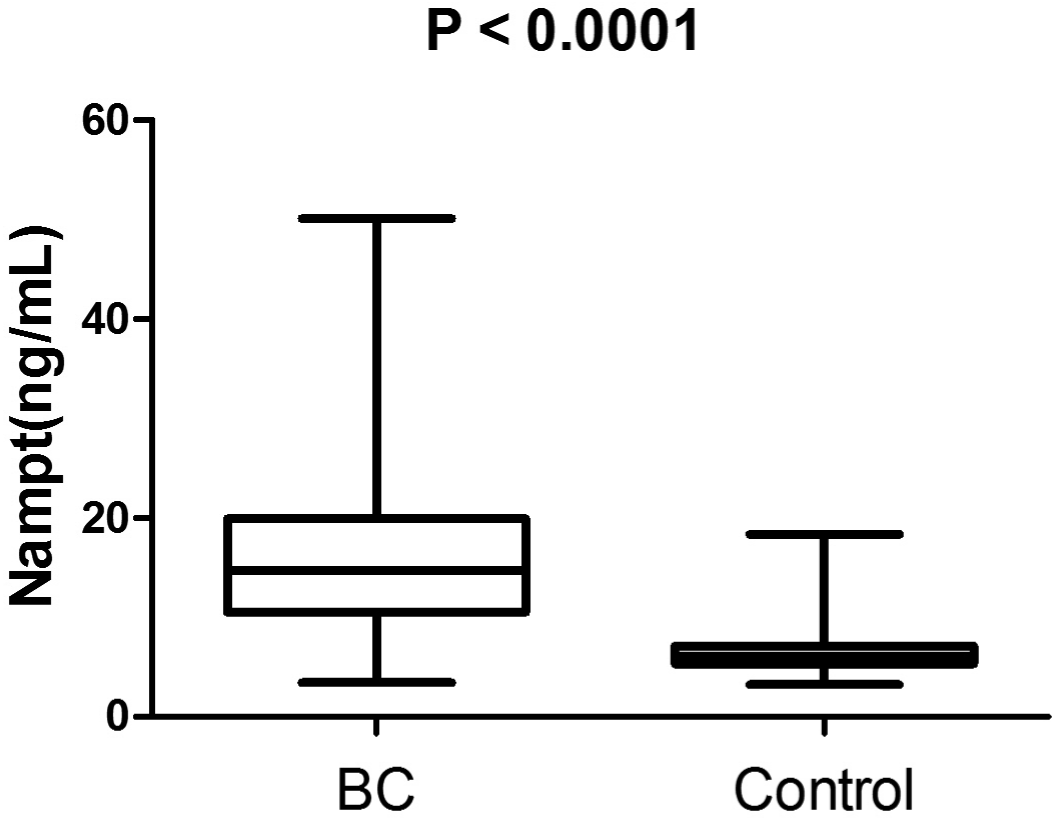
Comparison of nicotinamide phosphoribosyltransferase (Nampt) expression level between bladder cancer (BC) patients and controls.

The serum Nampt protein level in patients with BC (mean ± SD, 16.02 ± 7.95 ng/mL) was significantly higher than in the control group (*P* < 0.001) (6.46 ± 2.08) (Figure 1). The median serum Nampt protein level was 14.74 ng/mL (range 3.44-50.15). The below median and above median levels were respectively found in 66 (50.4%) and 65 (49.6%) patients.

**Figure 2:**
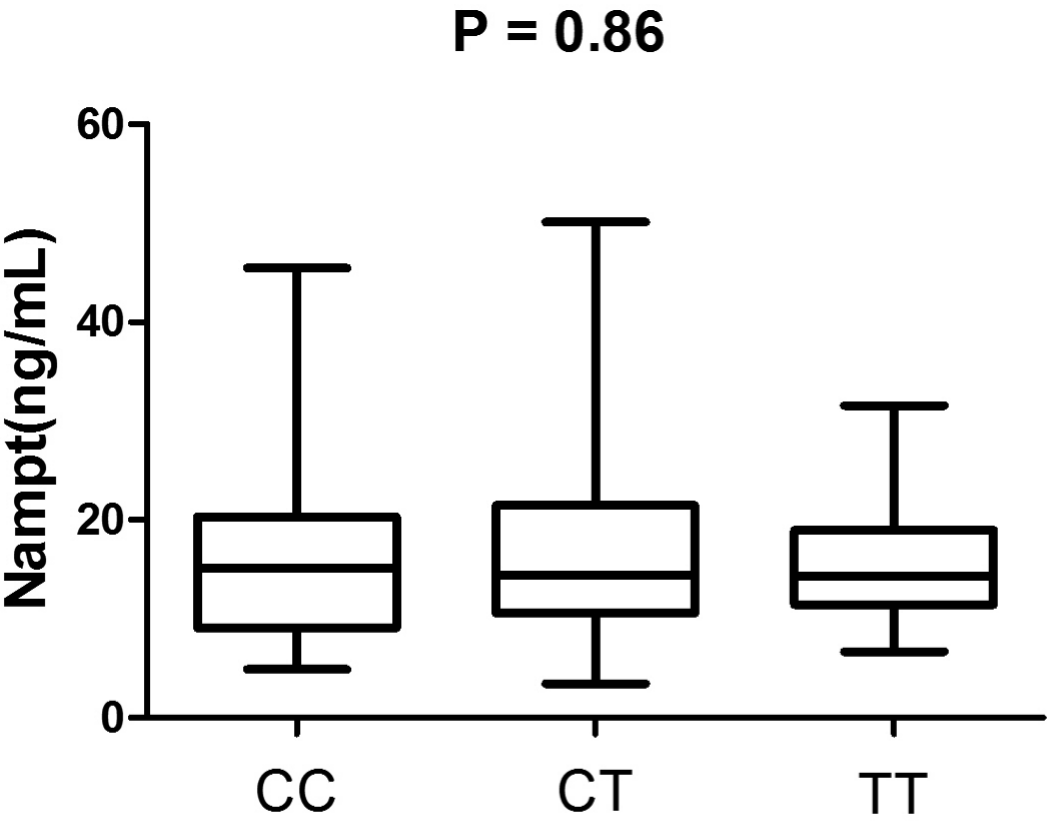
Comparison of nicotinamide phosphoribosyltransferase (Nampt) expression level among three *rs2505568* genotypes.

Of 131 BC patients genotyped for *rs61330082*, 39 were CC homozygous, 60 CT heterozygous, and 32 TT homozygous. All observed genotype frequencies were in agreement with the Hardy-Weinberg equilibrium expectations. One-way ANOVA analysis revealed no significant differences in serum Nampt level between the three genotypes (Figure 2).

### Relationship between serum Nampt protein level and clinical pathological features of patients with BC

No significant correlation in BC patients was observed between serum Nampt protein level and clinical pathological features, including age, sex, smoking status, tumor grade, invasiveness, metastasis, and recurrence (Table 2).

**Table 2:** The relationship between serum nicotinamide phosphoribosyltransferase (Nampt) and clinical pathological features of patients with bladder cancer

Pathological features	N (%)	Serum Nampt		Odds ratio (95%confidence interval)	*P* (χ^2^ test)
		≤14.74 ng/mL	>14.74 ng/mL		
**Age**					
≤64	57 (43.5)	24	33	1 (referent)	
>64	74 (56.5)	42	32	0.554 (0.276-1.114)	0.096
**Sex**					
Male	107 (81.7)	52	55	1 (referent)	
Female	24 (18.3)	14	10	0.675 (0.276-1.654)	0.389
**Smoking**					
No	54 (41.2)	27	27	1 (referent)	
Smoking index ≤400	36 (27.5)	19	17	0.895 (0.385-2.081)	0.796
Smoking index >400	41 (31.3)	20	21	1.050 (0.466-2.365)	0.906
**Grade**					
Low	60 (45.8)	28	32	1 (referent)	
High	71 (54.2)	38	33	0.760 (0.382-1.513)	0.434
**Invasiveness**					
Muscle-invasive	72 (55.0)	37	35	1 (referent)	
Non-muscle-invasive	59 (45.0)	29	30	0.094 (0.549-2.177)	0.799
**Relapse**					
No	90 (68.7)	48	42	1 (referent)	
Yes	41 (31.3)	18	23	1.460 (0.695-3.070)	0.317
**Metastases**					
No	111 (84.7)	55	56	1 (referent)	
Yes	20 (15.3)	11	9	0.804 (0.309-2.091)	0.654

### Prognostic value of serum Nampt level

**Figure 3:**
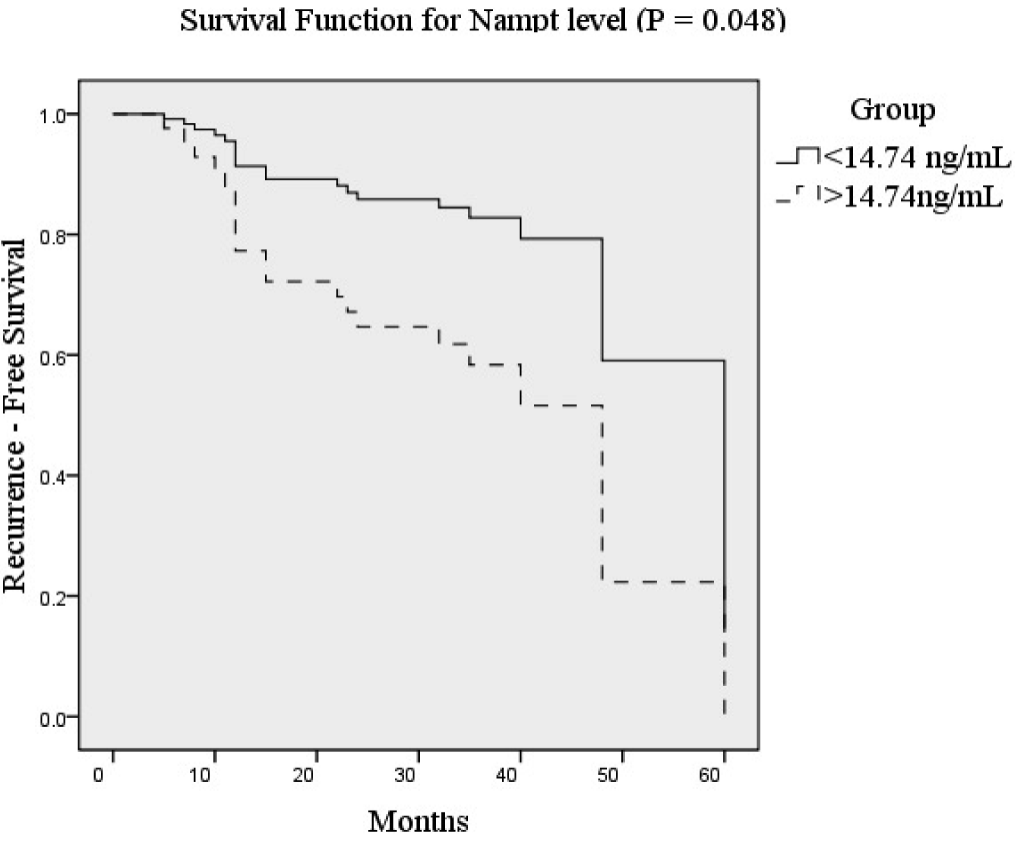
Kaplan-Meier recurrence-free survival curves for non-muscle-invasive bladder cancer patients based on serum nicotinamide phosphoribosyltransferase (Nampt) level.

Cox multivariable regression analysis showed that serum Nampt level was an independent prognostic marker of non-muscle-invasive BC, with higher level (>14.74 ng/mL) indicating shorter recurrence-free survival rate (hazard ratio = 2.85, 95% confidence interval = 1.01-8.06, *P* = 0.048) (Table 3). However, there was no significant association of serum Nampt level with RFS or OS in total BC patients and muscle-invasive BC patients (Figure 3).

**Table 3:** Association between nicotinamide phosphoribosyltransferase (Nampt) level and patient’s survival

Characteristics			Multivariate survival analysis						Univariate survival analysis					
			Recurrence-free survival*			Overall survival*			Recurrence-free survival			Overall survival		
		n	Hazard ratio	95% confidence interval	*P*	Hazard ratio	95% confidence interval	*P*	Hazard ratio	95% confidence interval	*P*	Hazard ratio	95% confidence interval	*P*
Total	≤14.74 ng/mL	66	1			1			1			1		
	>14.74 ng/mL	65	1.19	0.64-2.23	0.583	0.94	0.37-2.39	0.888	1.21	0.65-2.25	0.551	0.79	0.31-2.01	0.624
Muscle-invasive	≤14.74 ng/mL	37	1			1			1			1		
	>14.74 ng/mL	35	0.93	0.36-2.44	0.887	0.60	0.19-1.86	0.374	0.86	0.33-2.23	0.752	0.62	0.20-1.91	0.405
Non-muscle-invasive	≤14.74 ng/mL	29	1			1			1			1		
	>14.74 ng/mL	30	**2.85**	**1.01-8.06**	**0.048**	3.02	0.39-23.37	0.291	2.02	0.85-4.79	0.110	1.46	0.24-8.73	0.679

*Adjusted for age, sex, smoking status, tumor stage, and tumor grade.

†Significant results are marked in bold.

## Discussion

Our study showed that serum Nampt level may serve as a biomarker of BC and an independent prognostic marker of non-muscle-invasive BC, with higher serum Nampt level indicating shorter recurrence-free survival rate. The use of biomarkers can provide detailed insight into cancer progression and metastasis, leading to more accurate and patient-specific prognosis and surveillance, and it can improve patients’ quality of life by enabling the use of less-aggressive treatment options (18,19).

Human Nampt was originally characterized as a presumptive cytokine named pre-B-cell colony enhancing factor (PBEF) (20). Nampt was also claimed to function as an insulin-mimetic adipocytokine named visfatin (21). Nampt exists in 2 known forms, iNampt and eNampt. iNampt is involved in angiogenesis by activating the extracellular signal regulated kinase (ERK)1/2 pathway and inducing vascular endothelial growth factor and MMP2/9 production (22). Additionally, it induces the proliferation and capillary-like tube formation in human umbilical vein endothelial cells in a dose- and time-dependent manner (23). These findings suggest that iNampt might have pro-angiogenic activity and support the growth of some types of tumors. Additionally, a chemical screen performed to identify compounds that might affect mechanisms of cellular growth, survival, or death has yielded a potent small molecule inhibitor termed FK-866 (24). eNampt acts as a cytokine, independent of its enzymatic activity, and plays a major role in the regulation of immune responses (16).

Nampt is expressed throughout the body and secreted mainly by adipocytes and macrophages (25). Its activity has been shown to enhance cellular proliferation (26) and to tip the balance toward cell survival following a genotoxic insult (27). Also, Nampt plays a central role in controlling the circadian clock machinery by dictating the periodical oscillations of some of its key transcription factors (28). At the immune level, it has been shown to promote myeloid (29) and lymphoid (30) differentiation and to increase specific cytokine production (31). The complex regulatory network should in principle disqualify Nampt as a potential target for pharmacological intervention. Indeed, reduced NAD^+^ levels should affect the activity of several sirtuin members, causing multiple, and possibly unrelated, biological effects *in vivo* (32).

Other studies found that serum Nampt protein level was associated with tumor progression in patients with gastric cancer (33) and colorectal cancer (11). Nampt expression together with p53 expression was associated with shorter survival of glioblastoma patients (34). Nampt overexpression was also found to predict poor response to doxorubicin-based chemotherapy in breast cancer treatment (35). Our study contributed to this body of evidence by showing that serum Nampt level may be an independent prognostic marker of non-muscle-invasive BC, with higher serum Nampt level indicating shorter recurrence-free survival rate.

Our previous study showed that *rs61330082* in *NAMPT* promoter region may predict BC risk and prognosis (17). However in this study there was no significant difference between the three genotypes in serum Nampt level, which implies that *rs61330082* may not affect the bladder cancer risk and prognosis via influencing the serum Nampt level.

In conclusion, our study presents a novel finding that serum Nampt level may serve as biomarker of BC and an independent prognostic marker of non-muscle-invasive BC. Nampt may be therefore a promising target for BC treatment, but its exact function in BC remains to be evaluated.
